# A simple and effective method for the accurate extraction of kinetic parameters using differential Tafel plots

**DOI:** 10.1038/s41598-021-87951-z

**Published:** 2021-04-26

**Authors:** Prashant Khadke, Tim Tichter, Tim Boettcher, Falk Muench, Wolfgang Ensinger, Christina Roth

**Affiliations:** 1 Faculty of Engineering Sciences, University of Bayreuth, Universitaetsstr.30, 95440 Bayreuth, Germany; 2 Physical and Theoretical Chemistry, Free University of Berlin, Takustr. 3, 14195 Berlin, Germany; 3 Department of Materials- and Geoscience, Technische Universitaet Darmstadt, Alarich-Weiss-Str. 2, 64287 Darmstadt, Germany

**Keywords:** Fuel cells, Electrocatalysis, Batteries, Corrosion, Electrocatalysis, Electrochemistry, Fuel cells, Metal-organic frameworks

## Abstract

The practice of estimating the transfer coefficient ($\alpha$) and the exchange current (${i}_{0}$) by arbitrarily placing a straight line on Tafel plots has led to high variance in these parameters between different research groups. Generating Tafel plots by finding kinetic current, ${i}_{k}$ from the conventional mass transfer correction method does not guarantee an accurate estimation of the $\alpha$ and ${i}_{0}$. This is because a substantial difference in values of $\alpha$ and ${i}_{0}$ can arise from only minor deviations in the calculated values of ${i}_{k}$. These minor deviations are often not easy to recognise in polarisation curves and Tafel plots. Recalling the IUPAC definition of $\alpha$ , the Tafel plots can be alternatively represented as differential Tafel plots (DTPs) by taking the first order differential of Tafel plots with respect to overpotential. Without further complex processing of the existing raw data, many crucial observations can be made from DTP which is otherwise very difficult to observe from Tafel plots. These for example include a) many perfectly looking experimental linear Tafel plots (R^2^ > 0.999) can give rise to incorrect kinetic parameters b) substantial differences in values of $\alpha$ and ${i}_{0}$ can arise when the limiting current (${i}_{L}$) is just off by 5% while performing the mass transfer correction c) irrespective of the magnitude of the double layer charging current (${i}_{\mathrm{c}}$), the Tafel plots can still get significantly skewed when the ratio of ${i}_{0}/{i}_{c}$ is small. Hence, in order to determine accurate values of $\alpha$ and ${i}_{0}$, we show how the DTP approach can be applied to experimental polarisation curves having well defined ${i}_{L}$, poorly defined ${i}_{L}$ and no ${i}_{L}$ at all.

## Introduction

Tafel plots (TPs) are primarily used to obtain two important catalyst performance indicators namely the transfer coefficient $\alpha$, and the exchange current ${i}_{0}$. TPs are generated from the well-known Tafel equation, a case of the Butler–Volmer (BV) equation or Erdey-Gruz–Volmer equation^1^ at high overpotential, $\eta$. The BV equation with no mass transfer limitations is given as^2^

1 $${i}_{k}={i}_{0}\left[{e}^{{\alpha }_{A}f\eta }-{e}^{-{\alpha }_{C}f\eta }\right]$$

In Eq. (1), ${i}_{k}$ is the overall kinetic current, ${\alpha }_{A}$ and ${\alpha }_{C}$ are the experimentally obtained anodic and cathodic transfer coefficients corresponding to a single or multistep reaction,$f=F/RT$ and $\eta =E-{E}_{eq}.$ The variables $F$, $R,$ $T,$ $E$ and ${E}_{eq}$ are Faraday´s constant, the universal gas constant, the temperature, the operating potential and equilibrium potential, respectively. At larger $\eta$ one of the exponential terms in Eq. (1) becomes insignificant. Consequently, Eq. (1) reduces to,

2 $${i}_{k}={i}_{0}{e}^{\alpha f\eta }$$

3 $${\mathrm{ln}}\left({i}_{k}\right)=ln\left({i}_{o}\right)+\alpha f\eta$$

In Eqs. (2) and (3), the $\alpha$ is either ${\alpha }_{A}$ or ${\alpha }_{C}$ depending on the sign of the $\eta$. From a plot of ${\mathrm{ln}}\left({i}_{k}\right){\mathrm{vs}}\; \eta$, $\alpha$ is obtained from the slope of the curve in the Tafel regime and ${i}_{0}$ is obtained from its extrapolation to zero overpotential.

Since Erdey-Gruz and Volmer^3^ introduced the transfer coefficient in 1930s, its physical interpretation has changed significantly. A very detailed discussion on history and interpretation of transfer coefficients has been published elsewhere^4^. To briefly summarise, after Tafel generalized the current–voltage relation based on his hydrogen evolution reaction (HER) experiments on several different metals^5^, Erdey-Gruz and Volmer^3^ were the first to confirm the Tafel equation by applying laws of kinetics and introducing transfer coefficients. This became the theoretical basis of the Tafel equation. They suggested that at any overpotential the current (overall rate) can be expressed as the difference of the rate of forward reaction and backward reaction^3^.

4 $$J={Fk}_{2}{c}_{+}{e}^{-\alpha \left({E}_{R}+\eta \right)f}-{Fk}_{3}{c}_{H}{e}^{+\alpha \left({E}_{R}+\eta \right)f}$$

where $J$ is the current, ${k}_{2}$ and ${k}_{3}$ are the rate constants of neutralization of the hydrogen atom and the ionoziation of the hydrogen respectively, ${c}_{+}$ and ${c}_{H}$ are the surface concentrations of hydrogen ions and hydrogen and ${E}_{R}$ is the reversible potential^1^. Equation (4)  at higher overpotential becomes similar to the Tafel equation. Polanyi and Horiuti^6^ provided for the first time a physical meaning of the transfer coefficient from transition state theory and also suggested that Eq. (4) can be written in a form that a constant term (commonly termed as exchange current) can be multiplied to anodic and cathodic exponential terms resulting in an equation very similar to Eq. (1).

Frumkin^7–9^ proposed a corrected current-overpotential relation arguing that the reactant concentration, $c$ (at low currents) in Eq. (4) may not be the bulk concentration but rather the concentration at the outer Helmholtz plane. While studying the electroreduction of H^+^ ion, he found that the current measured at the electrode is independent of the H^+^ concentration. He suggested that the reaction occurs at the outer Helmholtz plane and the local concentration at this site is different than in the bulk. He also suggested that the driving force for the electron transfer must be the difference between the electrode potential and average potential at the outer Helmholtz plane rather than the potential difference between the electrode potential and the potential in the bulk solution.

For a multistep reaction (i.e. series of consecutive reaction steps) Parsons^10^ presented a methodology to determine the rate determining step (rds) from the Tafel slope with the help of a free energy diagram. Based on his work the transfer coefficient of a multistep reaction can be derived as^11^

5 $${\alpha }_{a}={n}_{p}+{n}_{q}{\beta }_{a}$$

6 $${\alpha }_{c}={n}_{r}+{n}_{q}{\beta }_{c}$$

where ${n}_{p}$ is the number of electrons transferred before the rds, ${n}_{q}$ is the number of electrons involved in rds, ${n}_{r}$ is the number of electrons transferred after the rds, ${\beta }_{a}$ and ${\beta }_{c}$ are the symmetry factors of the forward and backward reaction, respectively. The ${n}_{q}$= 0 when the chemical step becomes rate-determining and is equal to 1, when the electrochemical step is the rds.

Marcus^12,13^, while working on his solvent fluctuation model for electron transfer, showed that the symmetry factor $\beta$ (or transfer coefficient of a single step single electron reaction) is a function of overpotential as follows

7 $${\beta }_{c}=\frac{1}{2}\left(1-\frac{F\eta }{2{\lambda }_{m}}\right)$$

8 $${\beta }_{a}=\frac{1}{2}\left(1+\frac{F\eta }{2{\lambda }_{m}}\right)$$

where ${\lambda }_{m}$ is the reorganisation energy per mole. Hence, the modern view of Eq. (4) differs quite a lot from the time, when it was first published by Erdey-Gruz and Volmer^3^. However, recently Fletcher^11^ derived the same form of the BV equation (as in Eq. 1) from first principles stating that the outward form of the common BV equation can be still be used for Tafel analysis, but recognizing that the experimental Tafel slope can be a function of overpotential.

Referring to Eqs. (5)–(8), it is clear that one may observe (a) a single Tafel slope when the chemical step is rds (b) a double Tafel slope when there are two chemical steps as rds, and each rds exists at a certain overpotential range within the Tafel regime, (c) a single Tafel slope when the electrochemical step is rds and ${\lambda }_{m}$>> $F\eta$, (d) curved Tafel slopes when the electrochemical step is rds and ${\lambda }_{m}$ is small. Adding to these complexities, curved Tafel slopes can also be observed due to the concentration depletion at the surface of the elctrode^14^ and charging current influence^15^. Hence, before any mechanistic conclusions are made, it’s absolutely necessary to appropriately correct any data for concentration depletion and charging current or at least the distortion caused by these influences in the Tafel regime must be known prior to further analysis.

The effect of concentration depletion is generally corrected by an appropriate mass transfer correction (also called the Koutecky–Levich compensation). For example in rotating disk electrode (RDE) experiments using the following equation^16^,

9 $${i}_{k}=\frac{{i}_{L}i}{{(i}_{L}-i)}$$

where $i$ is the measured current and ${i}_{L}$ is the limiting current. The ${i}_{k}$ determined from Eq. (9) is used in Eq. (3) to generate the TPs. However, for gas evolving reactions such as for oxygen evolution reaction (OER) and hydrogen evolution reaction (HER), the Eq. (9) cannot be used directly because of an unknown ${i}_{L}$. In such cases, one resorts to a plot of ${\mathrm{ln}}\left(i\right){\mathrm{vs}}\; \eta$ for the extraction of kinetic parameters. When kinetics are sufficiently slow, the extraction of kinetic parameters from a plot of ${\mathrm{ln}}\left(i\right){\mathrm{vs}} \;\eta$ can be carried out with reasonable accuracy. The effect of the charging current is corrected by subtracting the polarisation curve without active species (blank measurements) from the polarisation curve with active species.

There are two critical issues often overlooked in the above-mentioned correction methods. Firstly, the limiting current used for mass transfer correction must be very precise^17^. In this context, it will be shown later that an offset of only 5% in the limiting current can easily result in curvature of TP leading to incorrect values of $\alpha$, even though the TPs in certain overpotential ranges may look linear with R^2^ > 0.99. In other words, a wide range of assumed limiting currents for mass transfer correction can still result in an apparent linear curve with R^2^ > 0.99. This becomes a critical issue for polarisation curves where the $i$ asymptotically reaches a limiting current. Secondly, the double layer correction by background subtraction is an oversimplification of the actual situation. Considering the current flow through a simple Randles-circuit where the impedance of charge double layer is placed in parallel to the impedance of charge transfer, the current flow through the double layer (${i}_{c}$) in absence and presence of a faradaic reaction become significantly different^18^. Therefore, ${i}_{c}$ measured in the blank measurements cannot be used to correct for double layer effects in the presence of faradaic reaction. At best, one can detect the negative effects of ${i}_{c}$ from Tafel plots but measurement of the magnitude of ${i}_{c}$ is very difficult. In an even worse scenario, when the ${i}_{c}\gg {i}_{o}$, this may introduce an additional slope (shown later) in the TPs allowing some researchers to incorrectly interpret this slope as the one due to the faradaic reaction.

Adding to the problem of the inaccurate mass transfer and charging current correction, there is a significant uncertainty in the extraction of $\alpha$ from the TPs due to the herein called straight-line-placement-problem. Because there is no clear possibility to define Tafel regime in many experiments, the $\alpha$ is found from the slope of an arbitrarily placed straight line on the TP. Or even sometimes the $\alpha$ is found from the placement of an arbitrary straight line on a curve which is in fact a curved TP. It is not uncommon in literature that for the same reaction-catalyst system different values of $\alpha$, and ${i}_{0}$ are reported. For instance, comparing the literature^19–22^, the ${i}_{0}$ reported for the oxygen reduction reaction (ORR) on Pt in 0.1 M KOH ranges from 10^–9^ A cm^−2^ to 10^–11^ A cm^−2^. While the reported values of $\alpha$ in these studies are between 0.86 to 1.06 (52–64 mV dec^−1^) at low $\eta$, it is between 0.122 to 0.23 (260–490 mV dec^−1^) at high $\eta$. Similarly, to name few examples, the trend of different Tafel slopes for a similar catalyst-reaction system is also common for OER^23–26^, HER^5,27–29^ and methanol oxidation reaction^30–32^. Such a wide range of values may certainly give rise to quite different mechanistic interpretations for the same reaction-catalyst system.

Henceforth the question remains, how can one estimate the relevant $\alpha$ and ${i}_{0}$ values? (a) when there is no defined rule for the placement of the straight line on the TP and (b) when there are several possible values of ${i}_{L}$ and ${i}_{c}$ that can lead to apparent straight lines in the TP. To some extent the answer lies in what graphs are used for extraction of $\alpha$ and thereby its interpretation. Within the framework of the IUPAC definition, the transfer coefficient is defined as^4^

10 $$\left| {\frac{1}{f}\frac{{d\left( {{\text{ln}}(i_{k} )} \right)}}{{d\eta }}} \right| = \alpha$$

Hence the differentiation of TP, herein termed as differential Tafel plot (DTP) gives $\alpha$. In this article, we discuss the advantages of using DTP (a plot of $\alpha$ vs $\eta$) over standard TP (a plot of ${\mathrm{ln}}\left({i}_{k}\right){\mathrm{vs}} \;\eta$) and how it can lead to accurate estimation of kinetic parameters for the uniformly accessible electrodes under steady state conditions such as for the rotating disk electrodes (RDEs).

Due to higher sensitivity of DTP over TP, it becomes easy to spot the negative effects of ${i}_{c}$ and false mass transfer correction. Although advantageous, this approach is adopted by very few research teams^17,33–35^. The sensitivity of DTP towards different electrode geometry, non-uniformly accessible electrodes and unsteady processes have been extensively studied^4,33,36^. In this article we focus on two critical ratios, the ratio of exchange current to limiting current (${i}_{0}/{i}_{L}$) and ratio of exchange current to double layer charging current (${i}_{0}/{i}_{c}$). It is shown how the Tafel slope and the Tafel range is sensitive to these ratios. From the shapes of DTP and on the basis of ratio ${i}_{0}/{i}_{L}$ and ${i}_{0}/{i}_{c}$, it is shown in which scenarios the TP leads to incorrect estimation of $\alpha$ and ${i}_{0}$. The exact interpretation of the value of $\alpha$ in terms of reaction mechanisms is not our main intent or goal and throughout the article our focus has been rather on the accurate extraction of kinetic parameters. In the first part of this paper, we use simulated curves to demonstrate the effect of the ${i}_{L}$ and the ${i}_{c}$, before we continue with experimental data (second part). In all of our experimental data, the $\alpha$ was found to be independent of overpotential for the large portion (up to *i* ≤ 0.9 ${i}_{L}$) of the polarisation curve. Hence the simulations were carried out considering the BV formalism as given in Eq. (1).

DTP is used to analyse the experimental data of ORR, ferricyanide reduction reaction and OER. These reactions served as exemplary case for polarisation curves with well-defined ${i}_{L}$, ill-defined ${i}_{L}$ and no ${i}_{L}$. With this, we advocate the use of DTP instead of TP for an accurate determination of kinetic parameters and to reduce scattered experimental data for similar reactions and catalyst systems.

## Results and discussions

### Tafel overpotential

The $|\eta |$ at which the Tafel region starts is the point in the TP where the contribution of the backward reaction is negligible compared to the forward reaction. This potential is herewith defined as Tafel-overpotential (TOP), ${\eta }_{TOP}$. Mathematically, the contribution of the backward reaction is never zero, but for practical approximation, it can be defined as the $\eta$, at which the kinetic current of the backward reaction contributes less than 1% to the overall kinetic current. In other words, ${i}_{k}/{i}_{k, A}$ or ${i}_{k}/{i}_{k, C}$ is greater than 0.99. Here ${i}_{k, A}$ and ${i}_{k, C}$ are the anodic and cathodic kinetic current. Hence, for an electrochemical reaction, a generalised TOP can be calculated by setting ${i}_{k}/{i}_{k, A}$ or ${i}_{k}/{i}_{k, C}$ = 0.99. Noting ${i}_{k, A}$ = ${i}_{0}{e}^{{\alpha }_{A}f\eta }$ and ${i}_{k}$ = ${i}_{0}[{e}^{{\alpha }_{A}f\eta }-{e}^{-{\alpha }_{C}f\eta }]$, then at $\eta$ = ${\eta }_{TOP}$, the ratio ${i}_{k}/{i}_{k, A}$ becomes,

11 $$\frac{{i}_{0}[{e}^{{\alpha }_{A}f{\eta }_{TOP}}-{e}^{-{\alpha }_{C}f{\eta }_{TOP}}]}{{i}_{0}{e}^{{\alpha }_{A}f{\eta }_{TOP}}}=0.99$$

12 $$\left| {\eta _{{TOP}} } \right| = \frac{{ln(100)}}{{f\sum \alpha }}$$

where $\sum {\alpha = \alpha _{A} }$+ ${\alpha }_{C}.$ At 25 °C, the Tafel region starts at ~ 118 mV when $\sum{\alpha}$ = 1 whereas it starts at ~ 59 mV when $\sum{\alpha}$ = 2. The ${\eta }_{TOP}$ calculated here may not resemble the experimental values where we have observed ${\eta }_{TOP}$ ranging from 0.3 to 0.65 V, largely due to the 200–400 mV of difference between the theoretical equilibrium potential and observed open circuit potential. Despite this, for the simulation purpose, the ${\eta }_{TOP}$ calculated according to Eq. (12) serves as a good reference point to discuss the features of DTP before and after ${\eta }_{TOP}$.

The DTP is generated after differentiating Eq. (1) with respect to $\eta$,

13 $$\frac{1}{f}\frac{d\left({{\mathrm{ln}}(i}_{k})\right)}{d\eta }=\frac{{\alpha }_{A}{e}^{{\alpha }_{A}f\eta }+{\alpha }_{C}{e}^{-{\alpha }_{C}f\eta }}{{e}^{{\alpha }_{A}f\eta }-{e}^{-{\alpha }_{C}f\eta }}$$

At $|\eta |$ > $\left|{\eta }_{TOP}\right|$, one of the exponential terms is negligible yielding the generalized expression as shown in Eq. (10). Hence the DTP, i.e. a plot of $\frac{1}{f}\frac{d\left({{\mathrm{ln}}(i}_{k})\right)}{d\eta }$ vs $\eta$ converges to a constant value beyond $\left|{\eta }_{TOP}\right|$. To determine the evolution of ${i}_{0}$ with respect to $\eta$, a plot of ${i}_{0}$ vs $\eta$ termed as the exchange current plot (ECP), can be generated from Eq. (2), once $\alpha$ from Eq. (10) is known. The ${i}_{0}$ is calculated only for $\left|\eta \right|>\left|{\eta }_{TOP}\right|$, so that Eqs. (2) and (10) are applicable.

**Figure 1:**
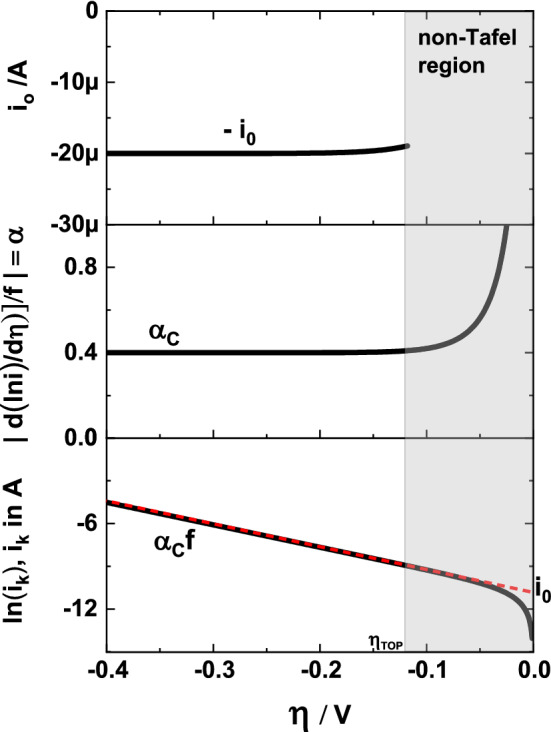
Theoretical TP (bottom), DTP (middle) and ECP (top). Assumed values: ${i}_{0}$ = 20 µA*,* ${i}_{L}$ = 1 mA*,* ${\alpha }_{A}$= 0.6*,* ${\alpha }_{C}$= 0.4*, T* = 21 °C*,* $f$=39.5 V^−1^*.*

Figure 1 shows from bottom to top, the TP and its corresponding DTP and ECP. The slope in the TP is equal to $\alpha f$ and at $\left|\eta \right|>\left|{\eta }_{TOP}\right|$, the DTP shows values of $\mathrm{\alpha }$. The shaded region highlights the non-Tafel region and is determined from Eq. (12).

The DTP in Fig. 1 is an ideal curve and asymptotically approaches to a constant value beyond $\left|{\eta }_{TOP}\right|$, a characteristic necessary for the determination of ${i}_{0}$ from ECP. We will show in the subsequent sections how ${i}_{c}$ and incorrect ${i}_{L}$ will distort the DTP and ECP behaviour, even when the TP appears to be unchanged.

### Effect of limiting current

**Figure 2:**
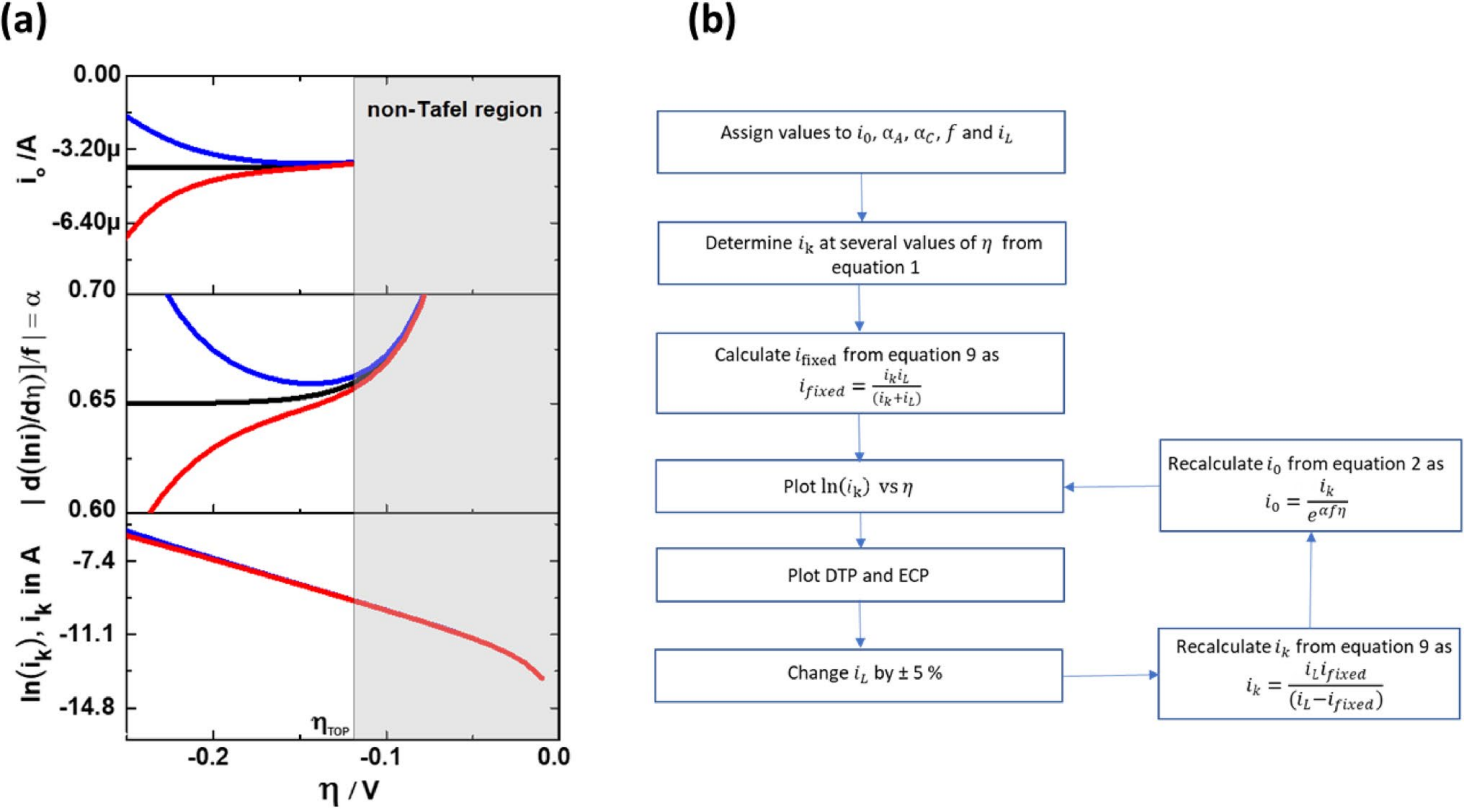
(**a**)Theoretically generated TP (bottom), DTP (middle) and ECP (top) for ${i}_{L}$=1.05 mA (red), ${i}_{L}$=1 mA (black) and ${i}_{L}$ = 0.95 mA (blue). Assumed values: ${i}_{0}$ = 4 µA, ${\alpha }_{A}$ = 0.35, ${\alpha }_{C}$ = 0.65, T = 21 °C, $f$ = 39.5 V^−1^. (**b**) Sequence for the calculation of ${i}_{k}$ when ${i}_{L}$ used for mass transfer correction changes by ± 5%.

The value of ${i}_{L}$ used for the calculation of ${i}_{k}$ in Eq. (9), is either observed from the polarisation curve or calculated from the Levich equation after experimentally determining the value of diffusion coefficient, viscosity and bulk concentration. According to the Levich equation, ${i}_{L}$ = $0.62nFA{D}^{2/3}{\upsilon }^{-1/2}{\omega }^{1/2}{C}^{*}$, where $n$ is the total number of electrons, $A$ is the geometric area, $D$ is the diffusion coefficient, $\upsilon$ is the viscosity, $\omega$ is the angular velocity and ${C}^{*}$ is the bulk concentration. Experimentally, small deviations in reading the ${i}_{L}$ value from the respective polarisation curve can occur, when the ${i}_{L}$ is not well-defined or when the $i$ asymptotically reaches a limiting current. Small deviations in the calculated value of ${i}_{L}$ from Levich equation can occur, when there is a small measurement error in the determined value of either diffusion coefficient, viscosity, or bulk concentration. The characteristics of TP, DTP and ECP for the two cases, when ${i}_{L}$ is either over- or underestimated by 5% are shown in Fig. 2a. To make simulation results comparable to experiments, the sequence as shown in Fig. 2b has been applied to calculate ${i}_{k}$.

Between the $\eta$ range of − 0.13 V to − 0.25 V, all calculated TPs are fitted with a straight-line equation and the fitting parameters are summarized in Table 1. Undoubtedly, the curve appears to be linear between − 0.13 to − 0.25 V in TPs because the R^2^ for all three TP is greater than 0.999. Nonetheless a different slope and hence different $\alpha$ is obtained for each ${i}_{L}$ value even though they are separated by only 5%, when using the over- or underestimated values. This imparts an error in estimation of $\alpha$ and eventually in the prognosis of the reaction mechanisms. Even though these errors cannot be easily inferred from TPs, the DTP is sensitive to them and shows significant differences, where there is a clear distinction between the three cases. Only when the ${i}_{L}$ is correct, the DTP converges to a constant $\alpha$ value beyond ${\eta }_{TOP}$, which is important for the calculation of ${i}_{0}$. From these plots we also see that when the value of ${i}_{L}$ is over- or underestimated, the $\alpha$ values in DTP and consequently ${i}_{0}$ in ECP are never approaching constant values with respect to $\eta$. Consequently, the same close to perfect linear stretch of the TP appears to be far from its ideal behavior in the DTP and ECP. The variance of $\alpha$ with respect to $\eta$ has been previously reported in the literature^37–39^ and this is interpreted in terms of reaction mechanism. However, referring to Fig. 2a, we would like to point out, that such a variation of $\alpha$ can also arise from the use of over- or underestimated values of ${i}_{L}$ in the calculation of ${i}_{k}$.

**Table 1:** Kinetic and fitting parameters corresponding to the TP of Fig. 2a. The $\upeta$ range for fitting is from − 0.13 to − 0.25 V.

*i*_*L*_ (mA)	R^2^	*i*_0_ (µA)	α	Slope	Intercept
1.05	0.99983	4.46	0.63	24.86	12.32
1.0	1.00000	4.00	0.65	25.61	12.44
0.95	0.99978	3.44	0.675	26.61	12.58

#### Tafel range with mass transfer correction

When the polarisation curve is mass transfer corrected, a substantial Tafel range can be easily obtained for slow reactions since the limiting current appears only at large overpotentials. However, for fast reactions, the Tafel region is experimentally observed only when ${i}_{L}$ in the polarisation curve appears at $|\eta |$>$\left|{\eta }_{TOP}\right|$. In this case, the usable range of $\eta$ for Tafel analysis, $\left|{\eta }_{TA}\right|$ can be found by considering the ${i}_{0}/{i}_{L}$ ratio and $\left|{\eta }_{TOP}\right|$. We can set $i=0.95{i}_{L}$ when it is assumed that Tafel analysis can be performed on polarisation curve until the current reaches 95% of the ${i}_{L}$.Then substituting $i=0.95{i}_{L}$ and ${i}_{k}={i}_{0}{e}^{\alpha f\eta }$, in Eq. (9) we get (full derivation in supplemental information),

14 $$\eta =\frac{{\mathrm{ln}}(19\; {i}_{L}/{i}_{0})}{\alpha f}$$

This is the $\eta$ at which the measured current is 95% of ${i}_{L}$. Considering Eqs. (12) and (14), the usable range of $\eta$ for Tafel analysis is,

15 $$\left| {\eta _{{TA}} } \right| = \left| {\frac{{{\text{ln}}(19\;i_{L} /i_{0} )}}{{\alpha f}}} \right| - \left| {\eta _{{TOP}} } \right|$$

**Figure 3:**
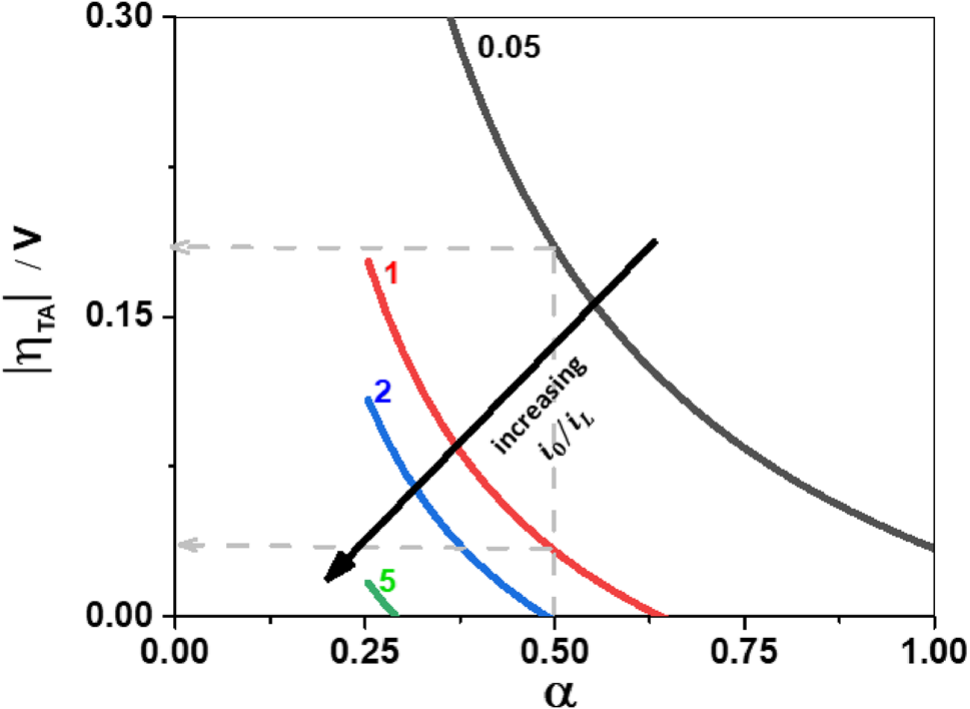
The usable potential range for Tafel analysis for the mass transfer corrected polarisation curves, when ${i}_{0}/{i}_{L}$= 0.05 (black), ${i}_{0}/{i}_{L}$= 1.0 (red), ${i}_{0}/{i}_{L}$= 2 (blue) and ${i}_{0}/{i}_{L}$= 5 (green). Example: at $\mathrm{\alpha }$ = 0.5, the Tafel behaviour is seen for a potential range of 185 mV and 30 mV when ${i}_{0}/{i}_{L}$ is equal to 0.05 and 1.0, respectively.

In Fig. 3, the usable range of $\eta$ for Tafel analysis is shown for various scenarios. When ${i}_{0}/{i}_{L}$= 5, few mVs are available only for low $\alpha$ values, hence Tafel analysis is not recommended. When the $\alpha$ > 0.5, also a ${i}_{0}/{i}_{L}$ ratio of 2 is not suitable for Tafel analysis. Only when the ${i}_{0}/{i}_{L}$< 0.05, Tafel analysis can be performed for all values of $\alpha$. Hence Tafel analysis for very fast reactions such as the hydrogen oxidation reaction (HOR) on Pt in acidic media might be difficult. The HOR on Pt in acidic media is so fast that the diffusion limited current in an RDE set up is observed already at small overpotential^40,41^. According to HOR studies on microelectrodes^42^, the ${i}_{0}$ for the HOR is between 27 and 80 mA·cm^−2^. In HOR studies on an RDE, the ratio of ${i}_{0}/{i}_{L}$ would be between 9–27 considering ${i}_{L}$= 3 mA·cm^−2^^41^. Hence, unless ${i}_{0}/{i}_{L}$ is reduced to less than 2 (assuming $\alpha$ = 0.5^41,43^) by experimental design, there will be no visible range of $\eta$ for Tafel analysis. Thus, irrespective of the $\alpha$ value, the foremost criteria for the polarisation curves with mass transfer correction is that the ratio ${i}_{0}/{i}_{L}$ should be less than 5 for any Tafel analysis to be conducted. This ratio becomes more and more restrictive as the $\alpha$ value increases.

#### Tafel range without mass transfer correction

For some experiments like HER and OER, the limiting currents are not easily observed and hence using Eq. (9) mass transport correction cannot be performed. In these cases, one resorts to extraction of kinetic parameters from a plot of ${\mathrm{ln}}\left(i\right)\mathrm{vs }\;\eta$ instead of ${\mathrm{ln}}\left({i}_{k}\right)\mathrm{vs }\;\eta$. This is, in general, a risky approach, however, in some cases where the ${i}_{0}/{i}_{L}$ is below a critical value, $\alpha$ and ${i}_{0}$ can be still extracted with reasonable accuracy. Rearranging Eq. (9), substituting ${i}_{k}={i}_{0}{e}^{\alpha f\eta }$ and taking the natural logarithm, the Eq. (9) reduces to (full derivation given in supplemental information),

16 $${\mathrm{ln}}\left(i\right)=ln\left(\frac{{i}_{o}{i}_{L}}{{i}_{L}+{i}_{0}{e}^{\alpha f\eta }}\right)+\alpha f\eta$$

Equation (16) is only valid for $|\eta |$ > $\left|{\eta }_{TOP}\right|$. A plot of ln $(i)$ vs $\eta$ is mathematically never linear by default, but approximates to linear curve when ${i}_{L}>>{i}_{0}{e}^{\alpha f\eta }$. When ${i}_{L}>>{i}_{0}{e}^{\alpha f\eta }$ , Eq. (16) in principle reduces to the Tafel equation. Setting ${i}_{L}$ to be at least 100 times greater than ${i}_{0}{e}^{\alpha f\eta }$, the suitable range of ${i}_{0}/{i}_{L}$ for the Tafel analysis can be found. For example, when ${i}_{L}= 100\cdot {i}_{0}{e}^{\alpha f\eta }$, then (full derivation given in Supplementary Information),

17 $$\eta =\frac{{\mathrm{ln}}({i}_{L}/100\;{i}_{0})}{\alpha f}$$

Subsequently, the usable range of $\eta$ for Tafel analysis is,

18 $$\left|{\eta }_{TA}\right|=\frac{{\mathrm{ln}}({i}_{L}/100\;{i}_{0})}{\alpha f}-\left|{\eta }_{TOP}\right|$$

**Figure 4:**
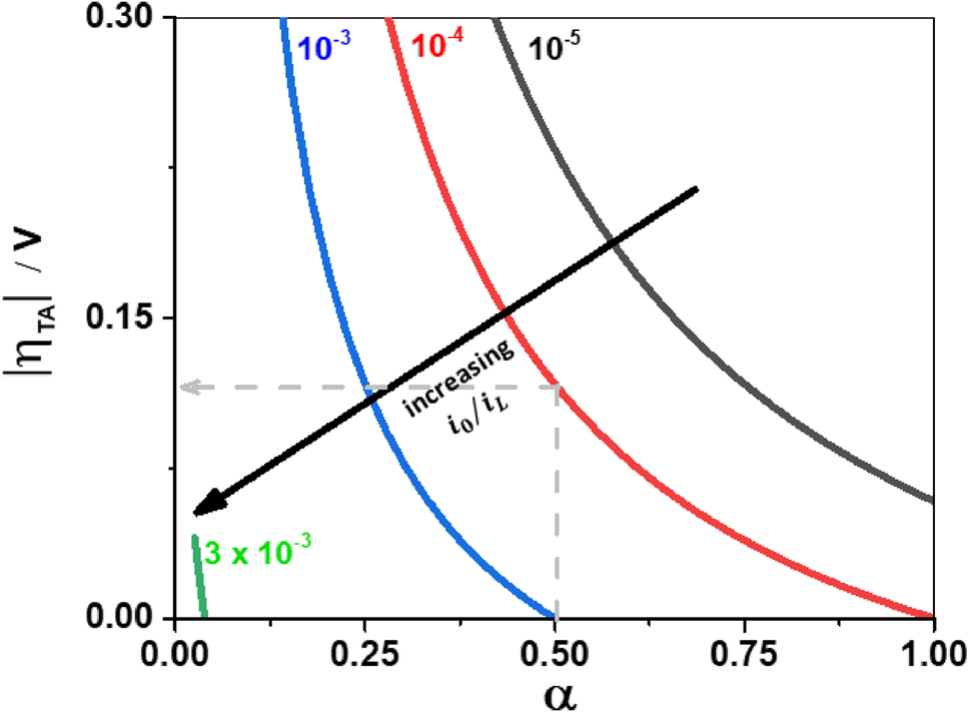
The usable potential range for Tafel analysis for the polarisation curves without mass transfer correction, when $i_{0} /i_{L} { }$ = 10^–5^ (black), $i_{0} /i_{L} { }$ = 10^–4^ (red), $i_{0} /i_{L} { }$ = 10^–3^ (blue) and $i_{0} /i_{L} { }$ = 3. 10^–3^ (green). Example: at $\alpha$ = 0.5, the Tafel behaviour is seen for a potential range of 115 mV when $i_{0} /i_{L} { }$ is equal to 10^–4^. Subsequently no usable potential range at $\alpha$ = 0.5 is observed for Tafel analysis when $i_{0} /i_{L}$ ≥ 10^–3^.

Similar to Fig. 3, in Fig. 4, the usable range of ${ }\eta$ for Tafel analysis is shown for various scenarios, except here the polarization curves are not mass transfer corrected. At a given $\alpha$, the $\eta_{TA}$ reduces as $i_{0} /i_{L}$ is increased. This graph shows that when $i_{0} /i_{L}$ > 0.003, the plot of $\ln \left( i \right)\;{\text{vs }}\eta$ is not suitable for extraction of kinetic parameters for any value of $\alpha$. At ${\upalpha }$ = 0.5, the $i_{L}$ should be at least 1000 times larger than $i_{0}$ to observe any potential region for Tafel analysis. Only when $i_{0} /i_{L}$ < 10^–4^, the kinetic parameters can be determined with reasonable accuracy from a plot of $\ln \left( i \right)\;{\text{vs }}\eta$ for all $\alpha$ values. Here the conditions for extracting kinetic parameters are much more restrictive compared to Fig. 3. In this sense, the plot of $\ln \left( i \right)\;{\text{vs }}\eta$ is quite limited for the extraction of kinetic parameters and care must be practiced when it is assumed that $i \sim i_{k}$ at $\left| \eta \right|$ > $\left| {\eta_{TOP} } \right|$.

### Effect of double layer charging current

**Figure 5:**
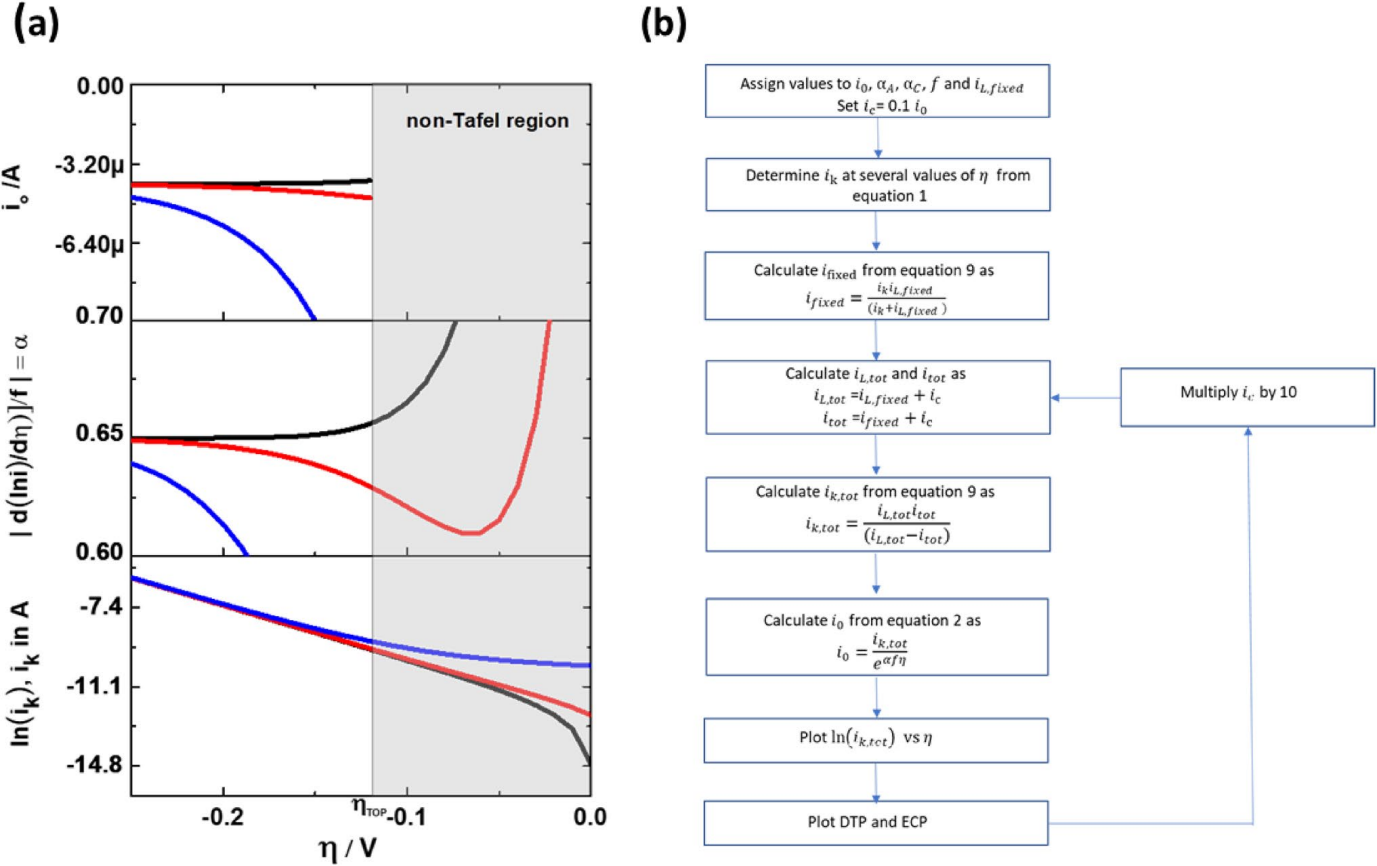
(**a**) Effect of double layer charging current on the TP (bottom), DTP (middle) and ECP (top) for $i_{0} /i_{c}$ = 10 (black), $i_{0} /i_{c}$ = 1 (red), $i_{0} /i_{c}$ = 0.1 (blue). For all curves $i_{L}$ = 10 mA, $i_{0}$ = 4 µA, $\alpha_{A}$ = 0.35, $\alpha_{C}$ = 0.65, *T* = 21 °C and $f$ = 39.5 V^−1^. (**b**) Sequence for calculating $i_{k}$ under the influence of double layer charging current.

When measuring the polarisation curve via linear sweep voltammetry, always a non-faradaic current flows along with the faradaic current. This non-faradaic current is due to charging or discharging of the double layer. Although the $i_{{\text{c}}}$ is relatively small at scan rates below 20 mV s^−1^, it can still have a significant effect on the progression of the DTP. In experiments, the measured linear sweep voltammetry current, $i_{{{\text{tot}}}}$ is approximately equal to the sum of $i$ and $i_{{\text{c}}}$. Similarly, the experimentally measured limiting current, $i_{L,tot}$ = $i_{L} + i_{{\text{c}}}$. Extraction of kinetic parameters is performed by considering $i_{{{\text{tot}}}}$ and *i*_*L,tot*_. Hence to mimic this scenario, in our simulations the $\alpha$ and $i_{0}$ has been determined from the modified kinetic current $i_{{{\text{k}},{\text{tot}}}}$. According to Eq. (9) this is given by $i_{{{\text{k}},{\text{tot}}}} = i_{L,tot} i_{tot} /(i_{L,tot} - i_{tot} )$. To generate $i_{{{\text{k}},{\text{tot}}}}$, the sequence as shown in Fig. 5b is adopted.

Figure 5a presents three scenarios with different ratios of $i_{0} /i_{c}$. Here, the $i_{L}$ is intentionally chosen high so that in all three cases $i_{L}$ >  > $i_{0}$ and $i_{L}$ >  > $i_{c}$. When $i_{0}$ is significantly larger than $i_{c}$, the DTP behaves normal, i.e. $\alpha$ and $i_{0}$ are constant with respect to $\eta$ at $\left| \eta \right|$> $\left| {\eta_{TOP} } \right|$. When $i_{0}$ is significantly smaller than $i_{c}$, the DTP is severely distorted making the estimation of $\alpha$ and $i_{0}$ difficult. The lower the $i_{0}$ value with respect to $i_{c}$, the larger becomes the distortion. Hence, it would be misleading to assume that $i_{c}$ has only a minor influence (for example at low scan rates) on the polarisation curves by comparing the relative magnitudes of $i$ (or $i_{L}$) to $i_{c}$. The most crucial ratio here is $i_{0} /i_{c}$. If $i_{c}$ is significantly larger than $i_{0}$, then irrespective of the magnitude of $i_{L}$, the DTP and ECP will get distorted in the Tafel region and, $\alpha$, $i_{0}$ cannot be estimated accurately. The negative effect of $i_{c}$ on TPs has been reported previously^15,44^, and from our TPs, some differences between the curves can also be seen, however they are not as pronounced as in DTP.

This result is crucial for catalysts with low catalytic activity and a comparably large double layer capacity, such as metal oxide catalysts, which are either supported or mixed with carbon material. As these materials often have low electron conductivities, the carbon powder is added to the catalyst layer to make it conductive^45–47^. However, while carbon does not increase the catalytic activity, it increases the double layer capacity. Therefore, when the carbon additive content with respect to the active material is increased, then $i_{0}$ would remain the same, while $i_{c}$ increases. In such cases, the $i_{c}$ may be significantly larger than $i_{0}$ and there is a good chance that the values of $\alpha$ and $i_{0}$ estimated from TPs would be incorrect. In such cases, DTP will be a valuable tool to detect the distortion in the TPs due to *i*_*c*_ and identify the true Tafel range. Even though we have made simpler assumption that the $i_{c}$ to be independent of overpotential here, the benefits of DTP in detecting the distortion in TPs due to $i_{c}$ will be still applicable.

**Figure 6:**
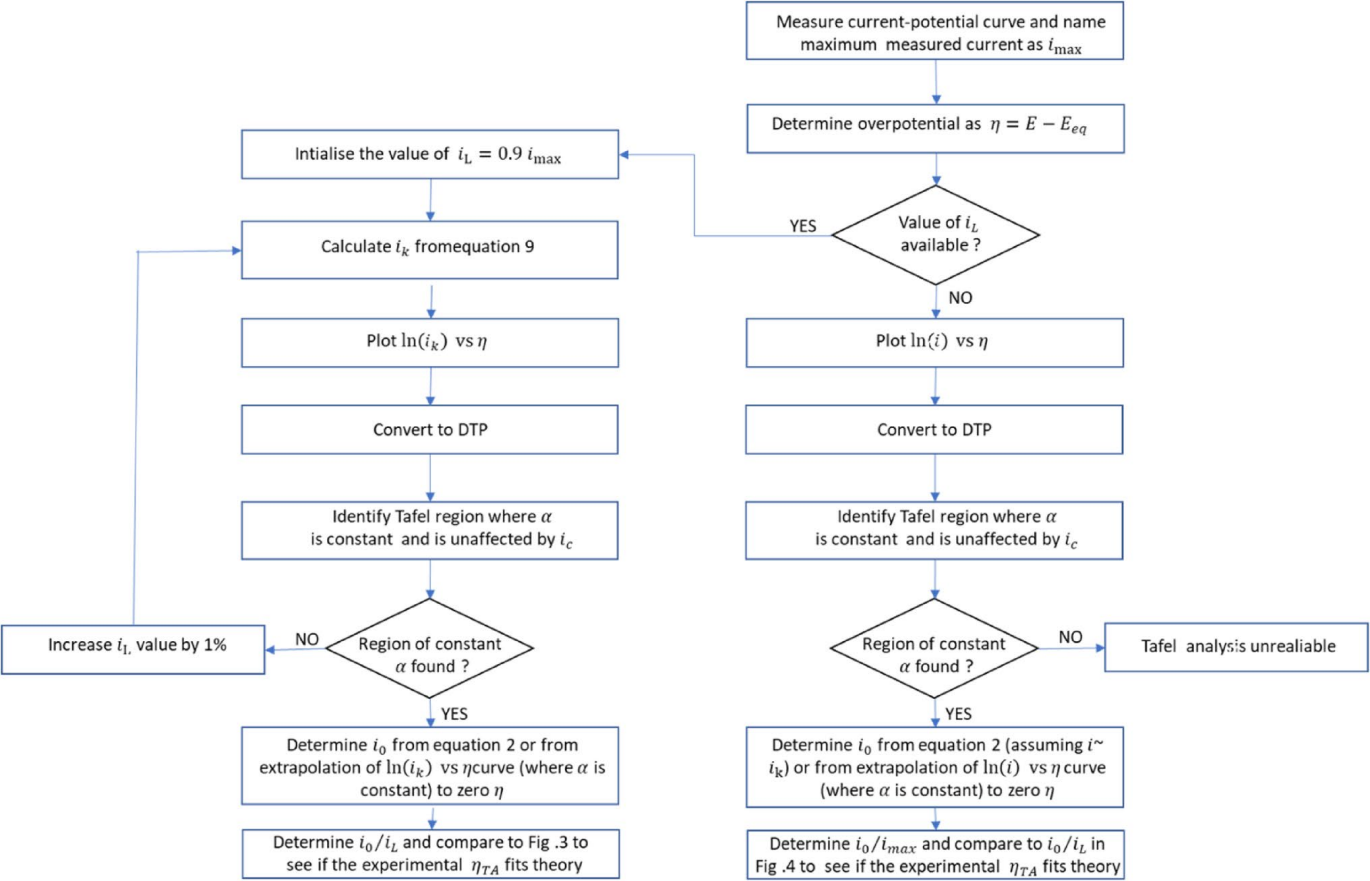
Scheme for extracting accurate kinetic parameters from the polarisation data.

We have seen that by combining the results of Figs. 3 and 4, at any given $\alpha$, it is possible to predict the whole range of $i_{0} /i_{L}$, where (a) Tafel analysis is not feasible (b) Tafel analysis is feasible, but only with mass transfer correction and (c) Tafel analysis is feasible with or without mass transfer correction. In addition, from Fig. 5a, we can identify the Tafel regime distorted by double layer charging current. This gives us some guidelines on how to process the experimental data. A recommended scheme is shown in Fig. 6.

### Experiments

Two difficulties in representation of DTP and ECP for the experimental data are worth mentioning here. First due to the small fluctuations of the measured polarization data and second due to the unknown value of $\eta_{TOP}$. In a DTP representation, the small fluctuations in polarization curves are amplified and hence the DTPs and corresponding ECPs are not smooth compared to their TPs and polarization curves. Therefore, fluctuations in DTP and ECP representation should not be necessarily considered as an indicator of unreliable data. For our measurements, without compromising the original shape and trend of curves, the data in DTP has been smoothened by moving average method. As discussed previously, the value of $i_{0}$ extracted from DTPs are valid only for $\left| \eta \right| > \left| {\eta_{TOP} } \right|$, i.e. when the $\alpha$ in the DTP approaches a constant value. In our experiments, since $\eta_{TOP}$ is unknown, an approach of $\alpha$ towards constancy has been taken as an indicator to define the data representation in ECP.

#### Polarisation curve with well-defined limiting current

**Figure 7:**
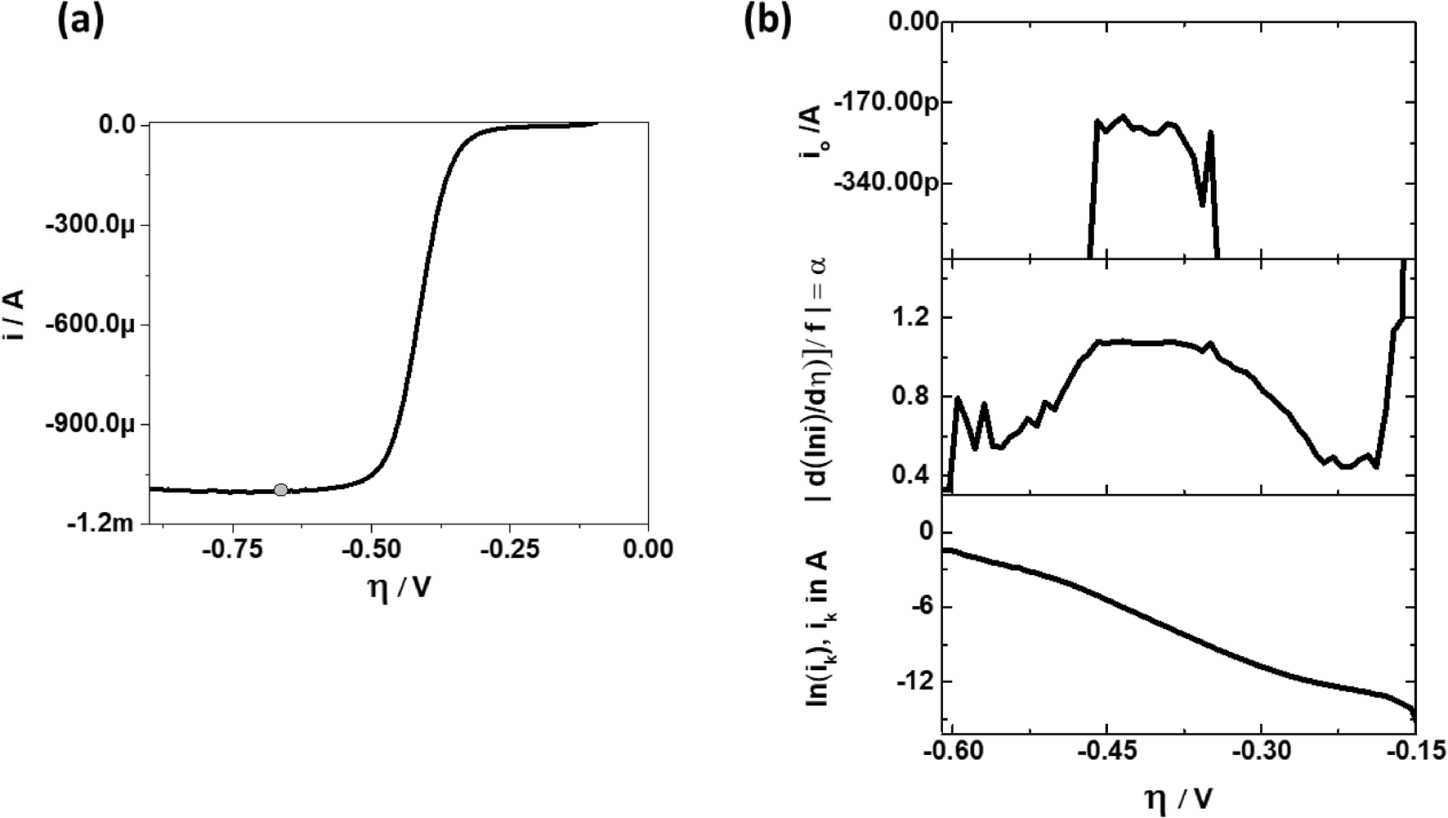
(**a**) ORR curves for GC disk coated with 20 µg_Pt_ cm^−2^. The ORR curves were measured at 10 mV s^−1^, 1600 RPM, 60 °C and 0.1 M KOH. The grey dot shows a point where the value of limiting current is defined. (**b**) The corresponding TP (bottom), DTP (middle) and ECP (top).

Figure 7a shows the ORR curve in alkaline media for glassy carbon (GC) disk coated with Pt/C particles. The loading of Pt is kept at 20 µg_Pt_·cm^−2^. With such high loadings, the Pt particles are uniformly distributed throughout the surface of the GC and hence the limiting current is well defined. The corresponding TP, DTP and ECP are presented in Fig. 7b. These plots are generated by assuming the $i_{L}$ value shown as grey dot in Fig. 7a.

Following the conventional approach for ORR on Pt in alkaline media, the TP can be interpreted as a curve consisting of two slopes^19–22^ or even three slopes if one considers the data between − 0.175 to − 0.275 V. Only from the corresponding DTP it becomes clear that the first slope (− 0.175 to − 0.275 V) is due to $i_{c}$, second Tafel slope (− 0.31 to − 0.45 V) is indicated by constant $\alpha$ and evidence for the presence of the third slope (− 0.5 to − 0.6 V) is rather weak. Although the shape of DTP is distorted due to the effects of $i_{c}$ at low overpotentials, its effects subdues at high overpotentials and it does not have significant impact on the estimation of $\alpha$ and $i_{0}$. The kinetic values extracted from the third slope are not of any practical use as the potentials are very close to limiting current region where the electrochemical device is unlikely to operate. Hence, relying on TPs alone can easily lead to unwarranted slope values.

Here we would like to stress again that BV formalism is a reasonable model to explain our experimental results because a clear distinction can be made between cases in a DTP, (a) when $\alpha$ appears to change with $\eta$ as a result of incorrect mass transfer correction, or (b) when $\alpha$ appears to change linearly with $\eta$ according to Eqs. (5)–(8)^11^. In the former case, the DTP will show a constant $\alpha$ at one particular value of $i_{L}$(used in the calculation of $i_{k}$) and at all other values of $i_{L}$ the $\alpha$ appears to change non-linearly with $\eta ,$ as already seen in Fig. 2a. In the latter case, the DTP will show a linear dependency of $\alpha$ on $\eta$ at one particular value of $i_{L}$, and at all other values of $i_{L}$ the $\alpha$ appears to change non-linearly with $\eta$. For our experimental data, after appropriate mass transfer correction we always observed the former case. Therefore, and for simplicity, only the BV formalism is considered for the analysis.

#### Polarization curves of slow reaction with ill-defined limiting current

**Figure 8:**
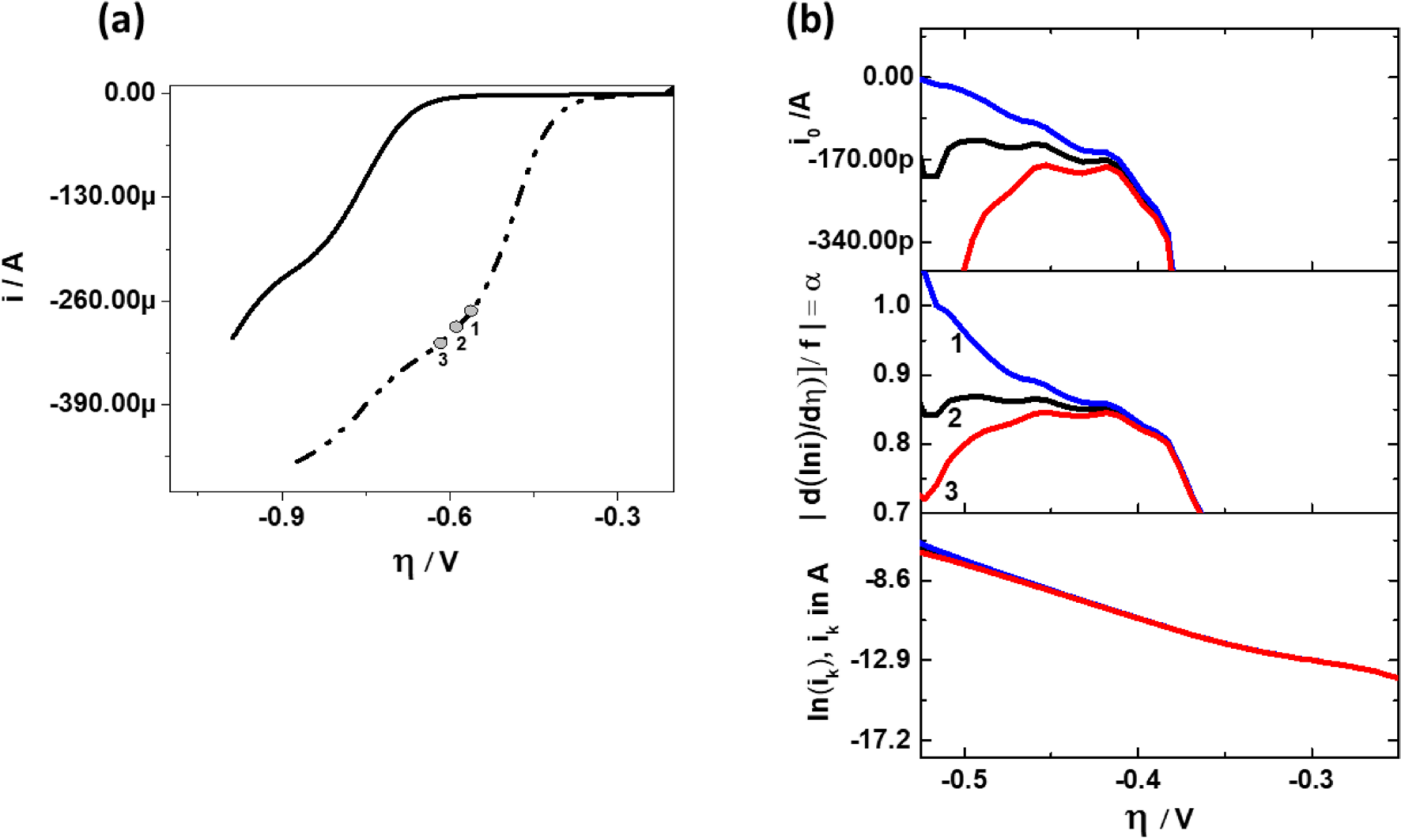
(**a**) ORR curves for GC disk (solid line) and GC disk coated with 5 µg_Pt_·cm^−2^ (dotted dashed line). The ORR curves were measured in 0.1 M KOH, 10 mV·s^−1^, 1600 RPM at 60 °C. The locations 1, 2, 3 are the positions at which $i_{L}$ is − 273µA, − 293µA and − 313µA respectively. (**b**) TP (bottom), DTP (middle) and ECP (top) for GC disk coated with 5 µg_Pt_ cm^−2^ assuming $i_{L}$ = − 273 µA (blue), $i_{L}$ = − 293µA (black) and $i_{L}$ = − 313 µA (red).

The DTP approach is especially useful for the interpretation of polarization curves having a poorly defined $i_{L}$. One such very prominent example for ill-defined limiting currents is the ORR in alkaline media at GC coated with low loading of Pt/C particles. Figure 8a compares the ORR curve of a GC coated with low loading of Pt/C with bare GC disk. The loading of Pt is 5 µg_Pt_·cm^-2^ and it is kept intentionally low to ensure some GC area remains uncovered. During polarization, Pt catalyzes ORR at low $\eta { }$ and at high $\eta$, additionally the underlying GC also catalyzes ORR^48^. This is due to the substantial activity of bare GC towards ORR at high $\eta$. Therefore, due to the background current of GC at high $\eta$, the observed limiting current for partially covered GC is not well defined. Had there been no catalytic activity from GC, the current would have reached a defined limiting current as commonly seen in acidic media^49,50^. The magnitude of the measured $i_{L}$ is proportional to the projected geometric area of the catalyst layer. For partially covered GC like the one described above, the projected geometric area of the catalyst layer is unknown and the $i_{L}$ with respect to the Pt nanoparticles is masked within the polarization curve. The platinum’s limiting current, however, is the value needed to find the kinetic parameters of Pt. It is shown in Fig. 8b how the features of the DTP can be exploited to obtain the kinetic parameters in this and similar cases. It shows from bottom to top TP, DTP and ECP of GC disk coated with 5 µg_Pt_·cm^−2^. In order to find the correct value of $i_{L}$ for the extraction of kinetic parameters, three arbitrary $i_{L}$ are chosen. This is shown as point 1, 2, 3 in Fig. 8a,b. These three $i_{L}$ values are used to calculate $i_{k}$ and generate the TP, DTP and ECP.

Unquestionably, there is visibly no difference for point 1, 2 and 3 in the respective TP. In fact, between − 0.4 and − 0.5 V, all the TPs presented in Fig. 8b have a slope with R^2^ > 0.999. Consequently, this feature makes it dangerous to extract kinetic parameters from TP and proceed further with mechanistic conclusions. In contrast to the TP, DTP and ECP are distinctly different and very sensitive to the three different cases. Only at point 2, where $i_{L}$ = − 293 µA, $\alpha$ becomes constant and hence this is the value of $\alpha$, that should be used for the determination of $i_{0}$. Please notice, that even though points 1 and 3 are quite close to point 2, there is a significant difference in $\alpha$ value in the DTP. This is in very good agreement with what has been shown previously in Table 1. Furthermore, in TP of Fig. 8b, depending on the overpotential range chosen for extraction of $\alpha$, the value of $i_{0}$ may differ by two orders of magnitude. Perhaps this is one of the causes for large variations in $i_{0}$ values in literature. Yet another possibility in large variation in $i_{0}$ is due to the calculation of overpotential. It can be calculated as $\eta = E - E_{eq}$ or $\eta = E - E_{OCV}$. For ORR on Pt electrodes, this difference is between 200 and 300 mV. This directly translates into two to three orders of difference in the extracted value of $i_{0}$ from the TP.

Compared to GC coated with 20 µg_Pt_·cm^−2^, here the $i_{c}$ distorts DTP even at higher $\eta$. The higher influence of $i_{c}$ in DTP for GC disk coated with 5 µg_Pt_·cm^−2^ is attributed to the effective decrease in the ratio $i_{0} /i_{c}$ because of the additional double layer capacity of uncovered GC.

#### Polarization curves of fast reaction with ill-defined limiting current

**Figure 9:**
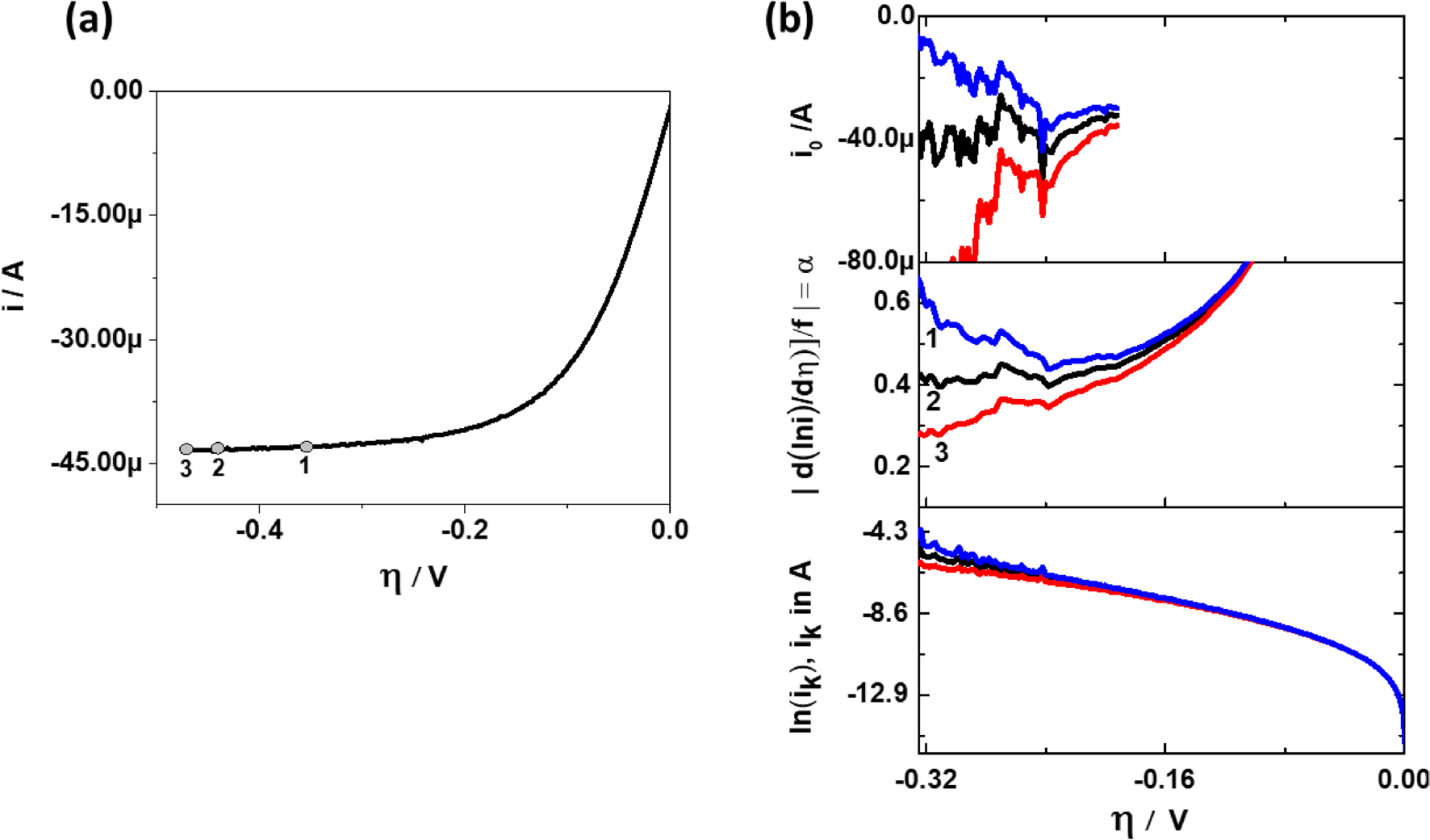
(**a**) Polarisation curve for potassium ferricyanide reduction on Au disk electrode. The locations 1, 2, 3 are the positions at which $i_{L}$ is − 43.0 µA, − 43.20 µA and − 43.52 µA respectively. The polarization curve was measured at a scan rate of 10 mV s^−1^, 4900 RPM and 21 °C in a solution consisting of 0.001 M K_3_Fe(CN)_6_, 0.001 M K_4_Fe(CN)_6_ and 0.1 M KCl. (**b**) TP (bottom), DTP (middle) and ECP (top) for potassium ferricyanide reduction on Au disk assuming $i_{L}$ = − 43.0 µA (blue), $i_{L}$ = − 43.2 µA (black) and $i_{L}$ = − 43.52 µA (red).

The ferricyanide reduction reaction on a gold electrode is a good example of a fast reaction. Figure 9a shows the polarisation curve of a ferricyanide reduction reaction measured on the Au disk electrode. In order to increase the mass transfer rate and thereby reduce the ratio of $i_{0} /i_{L}$, the polarisation curve has been measured at higher rotation rate of 4900 rpm. The $i_{L}$ is however, still not well defined and asymptotically reaches a constant value. It can be also seen that the so-called kinetic region indicated by the sharp exponential drop in potential at low current, is here absent. The shape of polarisation curve appears rather like a concentration-overpotential curve^51^ suggesting that kinetics in this example are indeed fast. Similar to Fig. 8, we have chosen here three arbitrary points (− 43.0 µA, − 43.20 µA and − 43.52 µA) in the limiting current region for the calculation of $i_{k}$ and thereafter generate TP, DTP and ECP. This is presented in Fig. 9b. The $\alpha$ and $i_{0}$ were found to be nearly constant for a small range of $\eta$, when $i_{L}$ = − 43.2 µA was used for the calculation of $i_{k}$. Even though the chosen values of limiting current vary by less than 1%, we see a sharp deviation in $\alpha$ and $i_{0}$ in DTP and ECP. This implies that for fast reactions, the kinetic parameters are extremely sensitive to selected value of the limiting current and due to large margin in the error, accurate estimation of $\alpha$ and $i_{0}$ is rather unlikely. In this respect, the DTP offers a robust methodology, wherein the $\alpha$ and $i_{0}$ can be estimated accurately by fine adjustment of limiting current. The obtained values of $\alpha$ and $i_{0}$ are about 0.4 and − 40 µA, respectively, when $i_{L}$ is chosen as − 43.2 µA. Thus, the ratio of $i_{0} /i_{L}$ ~ 1.0. From Eq. (15), the resulting $\eta_{TA}$ is circa 70 mV. This is roughly what can also be seen from the DTP of Fig. 9b confirming the predictions of Eq. (15). Hence, even when the exchange current is nearly equal to limiting current, a Tafel range is plausible and $\alpha$ and $i_{0}$ can be estimated accurately when the precise mass transfer correction has been carried out. Notice that due to the high value of $i_{0}$, the ratio $i_{0} /i_{c}$ is quite large and hence effects of $i_{c}$ are not visible in DTP in accord with the results presented in Fig. 5a.

#### Polarization curves without experimentally observed limiting current

**Figure 10:**
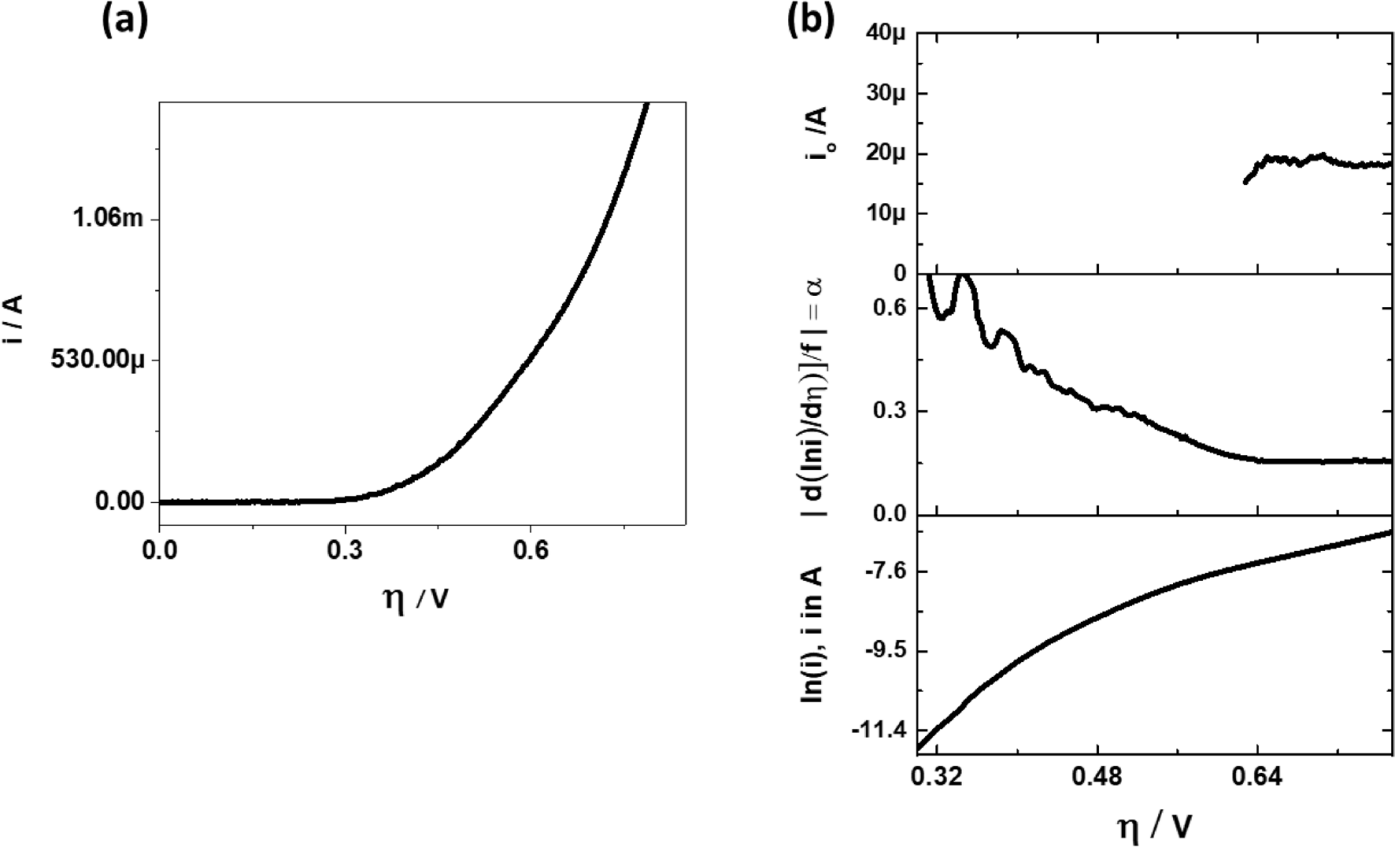
(**a**) Polarisation curve for OER on Pt disk electrode at 10 mV·s^−1^, 1600 RPM, 21 °C in 0.1 M KOH solution. (**b**) Respective TP (bottom), DTP (middle) and ECP (top).

The OER on a Pt disk is a classic example for an electrochemical reaction, in which no limiting current is observed for a wide range of $\eta$ (see Fig. 10a). In this case, one resorts to estimation of kinetic parameters from a plot of $\ln \left( i \right){\text{vs}}\: \eta$. Observing the evolution of $\alpha$ with respect to $\eta$ in the DTP, it can be found, whether the estimation of kinetic parameters from a plot of $\ln \left( i \right){\text{vs}} \:\eta$ is reasonable. In Fig. 10b the TP, DTP and ECP are generated without correcting the OER polarization data for mass transfer effects. Observing the trend in the TP, it might be tempting to place two straight lines between 0.60 to 0.75 V and 0.3 to 0.45 V and thereby conclude that two $\alpha$ values exist for OER. However, from DTP we can see that this is a false conclusion and leads to unwarranted mechanistic conclusions. The $\alpha$ in DTP and $i_{0}$ in ECP are nearly constant only at $\eta$ > 0.65 V. The presence of constant $\alpha$ and $i_{0}$ indicates that kinetic parameters from OER polarisation curves can be extracted without mass transfer correction. Hence with DTP we can confirm the presence of multiple $\alpha$ and determine if the polarisation curves require mass transfer correction before the extraction of kinetic parameters.

### Considerations for the design of the experimental set up

In the previous sections we have discussed the importance of the ratio $i_{0} /i_{L}$ and $i_{0} /i_{c}$. Depending on the $\alpha$, there is a maximum permissible value of $i_{0} /i_{L}$ in order to obtain a sufficiently large Tafel range. Consequently, certain measures can be considered when planning for the experiment. The experiments can be designed to achieve a reasonable range of $\eta$ for Tafel analysis. For a given catalyst, lowering the $i_{0}$ and increasing the mass transport will increase the accessible Tafel region. The $i_{0}$ can be lowered by reducing the ratio of microscopic area to geometric area, i.e. the catalyst layer should be as thin as possible. Among other possible methods, the mass transport can be increased by either increasing the rotation rate in an RDE set up or by utilizing a microelectrode. Although the mass transfer rate can also be increased by increasing the bulk concentration of reactant, this also proportionately increases the $i_{0}$^2^. Therefore, increasing the reactant concentration in some cases can essentially keep the ratio $i_{0} /i_{L}$ unchanged and may not extend the Tafel range for the analysis. Another possibility of increasing Tafel range is to reduce $i_{c}$ when feasible. If the increase in $i_{c}$ is caused by additional carbon/support material, the content of this carbon/support material may be reduced to keep the ratio $i_{0} /i_{c}$ high.

## Conclusion

The DTP approach is proposed as a simple and more precise alternative to TP for the accurate estimation of $\alpha$ and $i_{0}$. The presence of linear curve segment (even with R^2^ > 0.999) in TP cannot be considered as sufficient criteria for accurate estimation of $\alpha$ and $i_{0}$. For instance, we found rather weak evidence for the presence of second Tafel slope for ORR and OER on Pt and DTP revealed the presence of only one Tafel slope. The range, extent of linearity and presence of multiple Tafel slopes should be confirmed by DTP which ideally shows no to little variation of $\alpha$ with respect to $\eta$ for a given Tafel slope. This provides a clear quality criterion for assessing both measured and processed data. The role of $i_{L}$ in defining the Tafel slope has been often overlooked in literature. When the incorrect $i_{L}$ is chosen for mass transfer correction, the resulting $\alpha$ appears to be $\eta$ dependent. This behaviour is not obvious from either TP or polarisation curves. The DTP shows that $\alpha$ values are also severely distorted at small $\eta$ due to the charging of the double layer. When the ratio of $i_{0} /i_{c}$ < 0.1, the distortion extends into the Tafel region making it difficult to estimate $\alpha$ and $i_{0}$ accurately. The effect due to double layer charging current can be detected very well and the effect of mass transfer limitations can be corrected with the DTP method. The DTP method can be applied to polarisation curves having poorly defined limiting current (ex.: ORR on low loading Pt/C), no limiting current (ex.: OER on Pt disk) and well-defined limiting current (ex.: ORR on high loading Pt/C). Using these examples, we experimentally confirmed the capability of the DTP method to screen different catalyst materials in diverse electrochemical reactions, even under conditions which challenge conventional TP analysis. In summary, the DTP method shown in this article represents an accurate tool for interpreting polarisation data, allowing one to circumvent unwarranted mechanistic conclusions.

## Experimental section

The ORR experiments were carried out on in-house prepared GC disk (5 mm diameter) coated with Pt/C (60 wt% Pt, 40 wt% carbon) (Alfa Aesar) particles. A thin film of anion exchange ionomer was coated on Pt/C catalyst layer prior to recording ORR curves. The measurements were conducted in O_2_ saturated 0.1 M KOH (anhydrous, ≥ 99.95%, Sigma-Aldrich, ultrapure water Millipore 18.2 M ${\Omega }$ cm) at 60 °C. The OER experiments were carried out on Pt disk (3 mm diameter) in 0.1 M KOH at 21 °C. The Potassium Ferricyanide (III) (99%, Sigma-Aldrich) reduction was carried out on Au disk (3 mm diameter) in a deaerated solution consisting of 0.001 M K_3_(Fe(CN)_6_), 0.001 M K_4_(Fe(CN)_6_) and 0.1 M KCl (≥ 99.0%, Sigma-Aldrich)at 21 °C.

The Pt/C catalyst ink was prepared by adding 50 mg of 60 wt% Pt/C to 40 mL of deionized (DI) water (Millipore, 18.2 MΩ cm), which was sonicated for 20 min in an ice bath and was further diluted so as to obtain 0.1 μg_Pt_ in 1 μL of catalyst suspension. This suspension was sonicated each time in an ice bath for 10 min before the desired volume was dropped on the GC surface. The catalyst ink was dried under ambient conditions. Ionomer films of 0.5 μm thickness covering the catalyst coated GC disks were obtained by dropping 10 μL of ionomer solution, diluted accordingly from commercially obtained AS-4 ionomer solution (5 wt% solids in Isopropyl alcohol) supplied by Tokuyama, Japan. The density of recast ionomer film was assumed to be the same as A201 membrane (ρ = 1.06 g·cm^−3^) supplied by Tokuyama, Japan.

A standard three electrode RDE setup (Metrohm Autolab) was used for all electrochemical measurements. Mercury-mercurous oxide (0.165 V and 0.171 V *vs* SHE at 21 °C and 60 °C, respectively) (ALS Co. Ltd) in alkaline media and in-house prepared saturated Silver-Silver Chloride (0.199 V *vs* SHE at 21 °C) in 0.1 M KCl was used as reference electrode. A Pt coil was used as counter electrode for all measurements. The working electrodes were GC and GC coated with Pt/C for ORR, Pt disk for OER and Au disk for Ferricyanide reduction reaction. All the polarisation curves were measured by Gamry Reference-600 potentiostat (C3-Analysentechnik GmbH) at a potential sweep rate of 10 mV·s^−1^.

## Supplementary Information

Supplementary Information.

## List of symbol

$$\alpha$$: Transfer coefficient
${\alpha }_{A}$: Anodic transfer coefficient
${\alpha }_{C}$: Cathodic transfer coefficient
${\beta }_{c}$: Cathodic symmetry factor
${\beta }_{a}$: Anodic symmetry factor
${\lambda }_{m}$: Reorganisation energy per mole
$$\eta$$: Overpotential
$${\eta }_{TOP}$$: Tafel overpotential
${\eta }_{TA}$: Useable Tafel overpotential range
$\upsilon$: Viscosity of supporting electrolyte
$$\omega$$: Angular velocity
$A$: Geometric area of a disk electrode
${c}_{+}$: Surface concentration of hydrogen ions
${c}_{H}$: Surface concentration of hydrogen
${C}^{*}$: Bulk reactant concentration
$D$: Diffusion coefficient of reactant in liquid electrolyte
${E}_{OCV}$: Open circuit potential
$${E}_{R}$$: Reversible potential
$$F$$: Faraday’s constant
$$i$$: Measured current
${i}_{c}$: Double layer charging current
${i}_{k}$: Overall kinetic current
${i}_{k, A}$: Anodic kinetic current
${i}_{k, C}$: Cathodic kinetic current
$${i}_{L}$$: Limiting current
$${i}_{0}$$: Exchange current
$$J$$: Current
${k}_{2}$: Rate constant anodic
${k}_{3}$: Rate constant cathodic
${n}_{p}$: Number of electrons transferred before the rate determining step
${n}_{q}$: Number of electrons involved in rate determining step
${n}_{r}$: Number of electrons transferred after the rate determining step
$n$: Overall number of electrons transferred
$R$: Universal gas constant
R^2^: Coefficient of determination
$$T$$: Temperature

## Abbreviations

BV: Butler Volmer
DTP: Differential Tafel Plot
ECP: Exchange current plot
GC: Glassy carbon
HER: Hydrogen evolution reaction
HOR: Hydrogen oxidation reaction
OER: Oxygen evolution reaction
ORR: Oxygen reduction reaction
RDE: Rotating disk electrode
rds: Rate determining step
rpm: Rotation per minute
TOP: Tafel overpotential
