# Supplementary material for: A simple and effective method for the accurate extraction of kinetic parameters using differential Tafel plots
Source: Sci Rep. 2021 Apr 26;11:8974. doi: 10.1038/s41598-021-87951-z (PMC8076256; doi:10.1038/s41598-021-87951-z)
Supplement: Supplementary file 1 — Supplementary Information. [file 41598_2021_87951_MOESM1_ESM.docx]

Supplemental Information

A simple and effective method for the accurate extraction of kinetic parameters using differential Tafel plots

Prashant Khadke,*^[a]^ Tim Tichter,^[b]^ Tim Boettcher, ^[c]^ Falk Muench, ^[c]^ Wolfgang Ensinger, ^[c]^ Christina Roth^[a]^

[a] Dr. P. Khadke, Prof. C. Roth
Faculty of Engineering Sciences
University of Bayreuth
Universitaetsstr.30, D-95440 Bayreuth, Germany
E-mail: [prashant.khadke@uni-bayreuth.de](mailto:prashant.khadke@uni-bayreuth.de)

Christina.Roth@uni-bayreuth.de

[b] MSc. T. Tichter
Physical and Theoretical Chemistry
Free University of Berlin
Takustr. 3, D-14195 Berlin, Germany

E-mail: t.tichter@fu-berlin.de

[c] MSc. T. Tim Boettcher, Dr. F. Muench, Prof. W. Ensinger
Department of Materials- and Geoscience
Technische Universitaet Darmstadt
Alarich-Weiss-Str. 2,64287 Darmstadt, Germany

E-mail: [boettcher@ma.tu-darmstadt.de](mailto:boettcher@ma.tu-darmstadt.de)

[muench@ma.tu-darmstadt.de](mailto:muench@ma.tu-darmstadt.de)

wolfgang.ensinger@tu-darmstadt.de

Please refer to the manuscript for the equation numbers.

**Derivation of equation 10**

From equation 13

$$\frac{1}{f}\frac{d\left( {\ln(i}_{k}) \right)}{d\eta}=\frac{\alpha_{A}e^{\alpha_{A}f\eta}+\alpha_{C}e^{-\alpha_{C}f\eta}}{e^{\alpha_{A}f\eta}-e^{-\alpha_{C}f\eta}}$$

For the positive values of overpotential and at $\eta>\eta_{TOP}$

$e^{\alpha_{A}f\eta}$>>$e^{-\alpha_{C}f\eta}$

$$\frac{1}{f}\frac{d\left( {\ln(i}_{k}) \right)}{d\eta}=\alpha_{A}$$

**Derivation of equation 14**

substituting $i=0.95i_{L}$ and$i_{k}=i_{0}e^{\alpha f\eta}$, in equation 9 we get,

$$i_{0}e^{\alpha f\eta}=\frac{0.95{i_{L}}^{2}}{{(i}_{L}-0.95i_{L})}$$

$$i_{0}e^{\alpha f\eta}={19i}_{L}$$

$$e^{\alpha f\eta}={19 i_{L}}/{i_{0}}$$

taking natural logarithm,

$$\eta=\frac{{\ln(19 i_{L}}/{i_{0})}}{\alpha f}$$

**Derivation of equation 16**

Substituting $i_{k}=i_{0}e^{\alpha f\eta}$ in equation 9 we get

$$i_{0}e^{\alpha f\eta}=\frac{i_{L}i}{{(i}_{L}-i)}$$

$i_{L}i_{0}e^{\alpha f\eta}-ii_{0}e^{\alpha f\eta}$ = $i_{L}i$

$$i_{L}i_{0}e^{\alpha f\eta}= i(i_{L}+i_{0}e^{\alpha f\eta})$$

$$\frac{i_{L}i_{0}e^{\alpha f\eta}}{(i_{L}+i_{0}e^{\alpha f\eta})}=i$$

$$i=\frac{i_{L}i_{0}}{(i_{L}+i_{0}e^{\alpha f\eta})}e^{\alpha f\eta}$$

Taking natural logarithm

$$\ln\left( i \right)=ln\left( \frac{i_{o}i_{L}}{i_{L}+i_{0}e^{\alpha f\eta}} \right)+\alpha f\eta$$

**Derivation of equation 17**

Rearranging equation 9 we get,

$$\frac{1}{i}=\frac{1}{i_{k}}+\frac{1}{i_{L}}$$

When we set $i_{L}= 100\cdot i_{0}e^{\alpha f\eta}$, then $i_{L}= 100i_{k}$ , since $i_{k}{=i}_{0}e^{\alpha f\eta}$. Substituting in above equation we get,

$$\frac{1}{i}=\frac{1}{i_{k}}+\frac{1}{100i_{k}}$$

$$i=\frac{100i_{k}}{101}$$

Substituting this value $i$ of in equation 16,

$$\ln\left( \frac{100i_{k}}{101} \right)=ln\left( \frac{i_{o}i_{L}}{i_{L}+i_{0}e^{\alpha f\eta}} \right)+\alpha f\eta$$

or,

$$\ln\left( \frac{100i_{k}}{101} \right)=ln\left( \frac{i_{o}100i_{k}}{100i_{k}+i_{k}} \right)+\alpha f\eta$$

$$\alpha f\eta=\ln\left( \frac{100i_{k}}{101} \right)- ln\left( \frac{{100i}_{o}}{101} \right)$$

$$\eta=\frac{{ln(i_{L}}/{100i_{0})}}{\alpha f}$$
